# Analysis of Performance and Failure Modes of the IROC Proton Liver Phantom

**DOI:** 10.14338/IJPT-22-00043.1

**Published:** 2023-07-14

**Authors:** Hunter Mehrens, Paige Taylor, Paola Alvarez, Stephen Kry

**Affiliations:** 1 IROC Houston Quality Assurance Center, The University of Texas MD Anderson Cancer Center, Houston, TX, USA; 2 Department of Radiation Physics, The University of Texas MD Anderson Cancer Center, Houston, TX, USA; 3 The University of Texas MD Anderson Graduate School of Biomedical Science, Houston, TX, USA

**Keywords:** liver, phantoms, quality assurance, IROC

## Abstract

**Purpose:**

To analyze trends in institutional performance and failure modes for the Imaging and Radiation Oncology Core’s (IROC’s) proton liver phantom.

**Materials and Methods:**

Results of 66 phantom irradiations from 28 institutions between 2015 and 2020 were retrospectively analyzed. Univariate analysis and random forest models were used to associate irradiation conditions with phantom results. Phantom results included pass/fail classification, average thermoluminescent dosimeter (TLD) ratio of both targets, and percentage of pixels passing gamma of both targets. The following categories were evaluated in terms of how they predicted these outcomes: irradiation year, treatment planning system (TPS), TPS algorithm, treatment machine, number of irradiations, treatment technique, motion management technique, number of isocenters, and superior-inferior extent (in cm) of the 90% TPS isodose line for primary target 1 (PTV1) and primary target 2 (PTV2). In addition, failures were categorized by failure mode.

**Results:**

Average pass rate was approximately 52% and average TLD ratio for both targets had slightly improved. As the treatment field increased to cover the target, the pass rate statistically significantly fell. Lower pass rates were observed for Mevion machines, scattered irradiation techniques, and gating and internal target volume (ITV) motion management techniques. Overall, the accuracy of the random forest modeling of the phantom results was approximately 73% ± 14%. The most important predictor was the superior-inferior extent for both targets and irradiation year. Three failure modes dominated the failures of the phantom: (1) systematic underdosing, (2) poor localization in the superior-inferior direction, and (3) range error. Only 44% of failures have similar failure modes between the 2 targets.

**Conclusion:**

Improvement of the proton liver phantom has been observed; however, the pass rate remains the lowest among all IROC phantoms. Through various analysis techniques, range uncertainty, motion management, and underdosing are the main culprits of failures of the proton liver phantom. Clinically, careful consideration of the influences of liver proton therapy is needed to improve phantom performance and patient outcome.

## Introduction

The Imaging and Radiation Oncology Core’s (IROC’s) anthropomorphic phantoms are used as an end-to-end quality assurance test of radiation therapy delivery following the institution’s clinical workflow [1–3]. This includes phantoms used for proton therapy clinical trial quality assurance, the importance of which has increased as proton therapy use and availability have increased in the past 30 years. IROC currently has proton phantoms for 6 anatomical sites [4]: brain, head and neck [5], prostate, spine [6], lung [7–9], and liver [9]. The pass rates for these phantoms tend to lag behind those of their photon beam counterparts [10–12], so understanding how to improve these pass rates is essential towards not only helping clinics improve their clinical workflow, but also increasing enrollment in and adherence to National Cancer Institute–sponsored clinical trials for proton therapy.

The main benefit of proton therapy for liver malignancies, such as hepatocellular carcinoma and cholangiocarcinoma, is its ability to spare healthy liver tissue and nearby organs-at-risk (OARs) while achieving local control of the tumor [13, 14]. However, range errors due to relative stopping power uncertainty, in combination with motion, can exacerbate dose delivery errors especially for the proximal and distal ends of the proton beam [13, 14]. These limitations can be assessed by IROC’s proton liver phantom to see if clinics are achieving desired clinical goals and successfully managing the dosimetric challenges.

IROC’s proton liver phantom reflects the challenges for clinical proton therapy treatment of liver malignancies: there are 2 targets that must both receive the intended dose, the liver structure moves to present a moving target, and there are multiple different materials used that introduce different relative stopping powers that test range uncertainty. The purpose of this study was to evaluate the pass rate for the proton liver phantom, identify the common failure modes to help elucidate shortcomings of clinic’s processes, and thereby help improve proton phantom performance. Specifically, our analysis consists of various univariate and random forest modeling to predict outcome of the proton liver phantom and the variables of importance for that prediction. These findings can be directly translated into the clinic, thereby improving the treatment of patients.

## Materials and Methods

### Imaging and Radiation Oncology Core’s Phantom

IROC’s proton liver phantom contains 2 non-coplanar targets made of solid water (Gammex, Middleton, Wisconsin): primary target 1 (PTV1) is an oblong spheroid with a 2-cm diameter in the superior left quadrant. Primary target 2 (PTV2) is a sphere of diameter 3 cm in the inferior right quadrant. The liver, minus the targets, is made of blue water (Standard Imaging, Madison, Wisconsin) and an anteriorly located, single acrylic positioning rod. Outside of the liver, the phantom is a water-filled polyvinyl chloride shell. To provide realistic planning and delivery obstacles, the phantom includes 2 coplanar OARs made of polybutylene terephthalate rods that are left and right of the targets.

An acrylic motion table is used to introduce motion in the superior-inferior direction of approximately 1 cm. The motion is a clinical patient breathing trace and both targets move in unison. The accuracy of the delivered dose to both targets is measured with 2 double-loaded thermoluminescent dosimeters (TLDs) in each target. Two planes (coronal and sagittal) of GAFchromic film are also used for each target to assess planar dose distributions.

Institutions are instructed to deliver a treatment consistent with their clinical practice, with at least 95% of both targets receiving 6 GyRBE (relative biological effectiveness, taken as 1.1). The targets can be irradiated with 1 or 2 isocenters, based on the clinic’s workflow. Specifically for IROC’s proton liver phantom, relative stopping powers are provided for the outer shell, OAR polybutylene terephthalate rods, and acrylic positioning rod structure within the phantom for clinics to override. To pass, measured point dose (TLD) must agree with the treatment planning system (TPS) calculation within ±7% in each of the targets, and ≥85% of pixels must pass a 7%/4-mm gamma analysis on each of the 2 film planes for each target.

### Data Set

Proton liver phantom data were collected from 2015-22, culminating in 66 irradiations from 28 institutions. Fifteen values were extracted for each irradiation: overall phantom performance (pass/fail) status, average PTV1 TLD ratio (measured to calculated), average PTV2 TLD ratio, average of percentage of pixels passing gamma for both planes for PTV1, average of pixels passing gamma for both planes for PTV2, as well as irradiation year, TPS, TPS algorithm [15], treatment machine, number of irradiations, treatment technique, motion management technique (internal target volume (ITV), gating, tracking, none—ie, static irradiation), number of isocenters, and superior-inferior extent (in cm) of the 90% TPS isodose line for PTV1 and PTV2. Irradiation date was defined only based on year and considered as a categorical variable. Irradiation technique was broken down into scattered, uniform scanning, and pencil beam scanning. The TPS algorithm was defined as either pencil beam or Monte Carlo. Demographic data are shown in **Table 1**.

**Table 1.** Demographics of the proton liver phantom data.

**Category**	**Constituents**	**N (% of total samples)**	**Pass rate, %**
Treatment machine	Hitachi	4 (6.1)	50
	IBA	30 (45.5)	50
	Mevion	13 (19.7)	39
	Varian	13 (19.7)	69
	Other	6 (9.1)	50
Irradiation technique	PBS	44 (66.7)	61
	Scattered	15 (22.7)	27
	Uniform scanning	7(10.6)	43
TPS algorithm	Monte Carlo	26 (39.4)	62
	Pencil beam	40 (60.6)	45
TPS	Eclipse	20 (30.3)	40
	RayStation	30 (45.5)	60
	XiO	6 (9.1)	50
	Other	10 (15.2)	50
Motion management technique	Breath hold	11 (16.7)	82
	Gating	7 (10.6)	43
	ITV	43 (65.2)	44
	Static	5 (7.6)	60

**Abbreviations:** PBS, pencil beam scanning; TPS, treatment planning system; ITV, internal target volume.

Note: N is the total number contained in each category with the percentage of total samples in parenthesis, followed by the respective pass rate of that constituent.

In addition, proton liver failures were categorized by failure mode for each target. The list of failure modes included: (a) systematic overdose/underdose, (b) local dose error in an isolated area, (c) localization dose error in the superior-inferior direction (motion direction), (d) localization dose error in a nonmotion direction, (e) global error, for example, irregular dose distribution, (f) range error, for example, relative linear stopping power [16], and (g) combination errors when 1 error was insufficient to describe the failure of the phantom, for example, underdose with range error (a+f) or localization dose error in the direction of motion and nonmotion (c+d). When analyzing frequency of failure modes, the combination errors category was broken down into the individual error modes to highlight the most common errors found to contribute to overall failure of the phantom.

### Analysis

We first evaluated the relationship between treatment conditions and the performance (pass/fail rate) of the phantom by using univariate analysis (Levene test, independent *t* test, and Pearson χ^2^ test; IBM SPSS 24, Armonk, New York). The χ^2^ test was performed to determine statistical significance based on pass/fail between the continuous and categorical variables, with linear regression providing statistical significance on yearly trends of continuous variables. In addition, the Levene test and *t* test were used to determine equality of variance and means, respectively, between motion management techniques, TPS algorithms, pass/fail, and number of isocenters.

Further analysis was done by using random forest methods to predict pass/fail (classification), as well as PTV1 TLD ratio, PTV2 TLD ratio, and average gamma pass rate for PTV1 and PTV2 (regression), through the above parameters [10]. For all random forest analysis, missing data were imputed by using 100 trees for all random forest models, then upsampled to ensure a balanced data set between pass and fail. In addition, 4 hyperparameters (number of trees, number of variables split to randomly sample for each descending node, percentage of samples to train on, and minimum number of samples within the terminal nodes) were tuned to minimize out-of-bag error. These hyperparameters were tuned over the following hyperspace: number of trees (200-1000), number of variables split to randomly sample for each descending node (2-6), percentage of samples to train on (55%, 63.2%, 70%, 80%), and minimum number of samples within the terminal nodes (1-20). Since the random forest algorithm is self-validating through bootstrap aggregating, we split our data set into a training (70%) and a testing set (30%). The implementation of these random forest models was run 10 times to assess fluctuations within the data and results. Random forest was performed by using R (4.0.2) via the ranger and missRanger packages [17–19].

## Results

**Figure 1.**
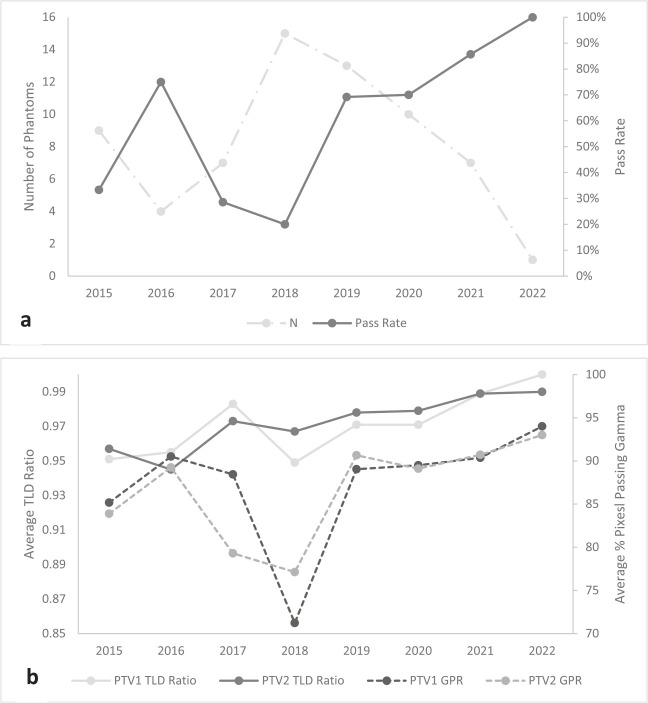
(a) Pass rates (solid orange) and number of irradiations (dashed blue) for the proton liver phantom through the years with an overall average pass rate of 51.5%. (b) Average TLD ratios and gamma pass rate values for the proton liver phantom for both targets through the years (solid blue = PTV1 TLD ratio, solid orange = PTV2 TLD ratio, dashed red = PTV1 gamma pass rate, dashed green = PTV2 gamma pass rate). Passing rates have fluctuated through the data cohort’s timeframe but recently (2019-22) the average has increased to approximately 74%. The TLD ratios and gamma pass rates have shown increase during the cohort’s timeframe; however, only PTV2 TLD ratio was statically significant. Abbreviations: GPR, gamma pass rate; PTV1, primary target 1; PTV2, primary target 2; TLD, thermoluminescent dosimeter.

The average pass rate of the entire IROC liver phantom cohort was 51.5%. Overall, the pass rate did not statistically significantly increase (*R*^2^ = 0.30, *P* = .20) during the complete timeframe of the data cohort; however, since 2019, the pass rate has been 74.2% (**Figure 1**). Furthermore, an increase in TLD:TPS ratio moved closer to unity for both PTV1 (*R*^2^ = 0.43, *P* = .11) and PTV2 TLD (*R*^2^ = 0.77, *P* = .01) by approximately 0.005 and 0.006 per year, respectively (increasing from 0.954 to 0.995 on average; **Figure 1b**). Gamma pass rates for both targets have increased over the cohort’s timeframe, with the exception of 2017-18, which saw a dip coinciding with the overall low pass rates observed. The overall trend is not statistically significant; however, an approximate 5% increase in both PTV1 and PTV2 gamma pass rates can be seen from 2015 to 2020 (**Figure 1b**).

**Figure 2.**
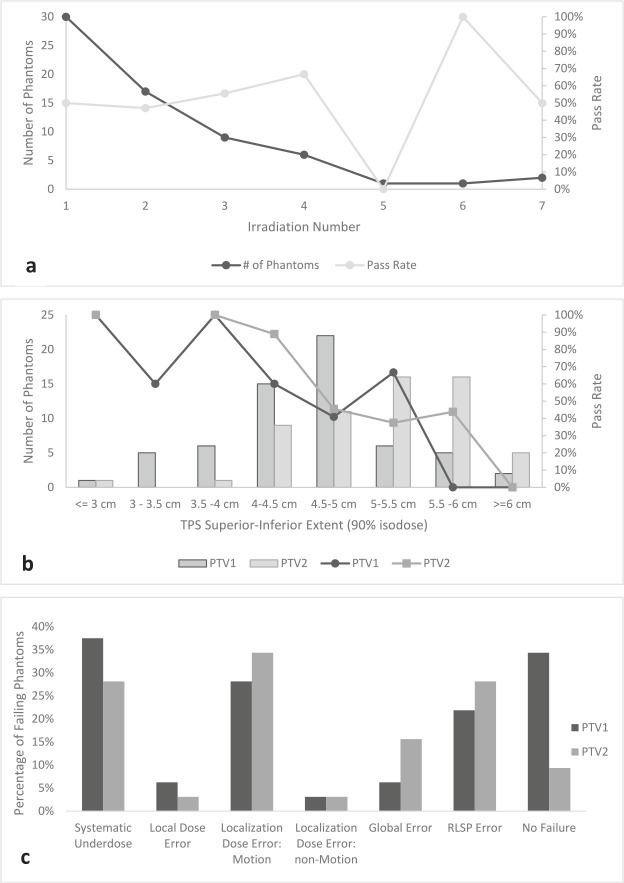
(a) The number of phantoms and their respective pass rates by irradiation number. The first and second irradiation by clinics tend to have a similar pass rate of approximately 50%, with the third irradiation and fourth irradiation having an increase of 5% and 15%, respectively. More than 1 analysis can be provided for an irradiation, that is, the clinic submits separate dose calculation algorithms for the same irradiation. (b) Number of phantoms and pass rate (blue = PTV1, orange = PTV2) of each target according to the superior-inferior extent of the TPS 90% isodose line. The larger the extent of the TPS target, the more likely that the phantom will fail. (c) Percentage of failing phantoms broken down by failure modes. The graph represents the combination category separated into its constituents. A total of 32 failures were analyzed regardless if they failed PTV1, PTV2, or both, hence why there is a no failure category, which implies that the phantom only failed one of the PTVs. The most common failure modes are systematic underdose, localization dose error in the motion direction (superior-inferior), and range error. Abbreviations: PTV1, primary target 1; PTV2, primary target 2; RLSP, relative linear stopping power; TPS, treatment planning system.

**Figure 2a** shows the pass rate and number of phantoms by number of times an institution irradiates the phantom. Institutions required as many as 7 attempts to successfully meet tolerance. The pass rate did not show any large increases as repeated attempts were made on the phantom. The phantom pass rate versus the size of the treatment field (as assessed by the superior-inferior extent of the TPS’s 90% isodose line) is shown in **Figure 2b**. Surprisingly, the larger the treatment field used to cover the target, the lower the phantom pass rate. Regression analysis showed this trend to be statistically significant for both PTV1 (*R*^2^ = 0.71, *P* = .008) and PTV2 TLD (*R*^2^ = 0.85, *P* = .003).

**Table 2** provides a breakdown of TLD ratios and gamma pass rate by machine, irradiation technique, TPS algorithm, TPS, and motion management technique. The χ^2^ analysis did not find any statistical significance between treatment parameters relative to pass/fail; however, Mevion machines (Mevion, Littleton, Massachusetts) had approximately 10% to 20% lower pass rate, scattered irradiation technique had approximately 15% to 35% lower pass rate, and gating and ITV motion management techniques had approximately 20% to 40% lower pass rates than other constituents within the respective category.

**Table 2.** Output parameters (mean ± SD) of the proton liver phantom data for constituents.

**Category**	**Constituents**	**Average PTV1 TLD ratio**	**Average PTV2 TLD ratio**	**Average PTV1 gamma pass rate**	**Average PTV2 gamma pass rate**
Treatment machine	Hitachi	0.98 ± 0.01	0.98 ± 0.01	94.9 ± 3.1	85.7 ± 11
	IBA	0.96 ± 0.03	0.97 ± 0.02	84.7 ± 16	85.4 ± 12
	Mevion	0.96 ± 0.05	0.97 ± 0.04	77.0 ± 17	81.4 ± 12
	Varian	0.98 ± 0.03	0.98 ± 0.01	90.7 ± 5.9	90.2 ± 6.4
	Other	0.96 ± 0.02	0.96 ± 0.03	82.7 ± 14	81 ± 15
Irradiation technique	PBS	0.97 ± 0.03	0.98 ± 0.02	87.3 ± 13	87.5 ± 10
	Scattered	0.95 ± 0.04	0.96 ± 0.03	74.8 ± 17	76.8 ± 13
	Uniform Scanning	0.97 ± 0.01	0.97 ± 0.01	90.4 ± 6.5	88.4 ± 5.8
TPS algorithm	Monte Carlo	0.98 ± 0.03	0.98 ± 0.02	88.3 ± 13	87.3 ± 11
	Pencil beam	0.96 ± 0.03	0.97 ± 0.02	82.5 ± 15	83.8 ± 11
TPS	Eclipse	0.96 ± 0.04	0.96 ± 0.02	80.0 ± 16	82.4 ± 12
	RayStation	0.98 ± 0.02	0.98 ± 0.02	88.3 ± 12	86.9 ± 12
	XiO	0.97 ± 0.01	0.96 ± 0.01	90.8 ± 6.9	89.5 ± 5.1
	Other	0.95 ± 0.04	0.97 ± 0.03	80.2 ± 20	83.1 ± 10
Motion management technique	Breath hold	0.97 ± 0.03	0.96 ± 0.02	91.7 ± 7.1	89.4 ± 9.0
	Gating	0.97 ± 0.02	0.98 ± 0.02	84.2 ± 13	81.9 ± 16
	ITV	0.96 ± 0.03	0.97 ± 0.02	83.5 ± 16	84.0 ± 12
	Static	0.96 ± 0.04	0.98 ± 0.03	81.1 ± 18	90.6 ± 5.4

**Abbreviations:** PTV1, primary target 1; TLD, thermoluminescent dosimeter; PTV2, primary target 2; PBS, pencil beam scanning; TPS, treatment planning system; ITV, internal target volume.

**Table 3** provides results for the Levene test and *t* test between pass-fail, Monte Carlo–pencil beam TPS algorithms, all motion management techniques, and number of isocenters for extent, TLD ratios, and gamma pass rate for both targets. Most notably, larger coverage of the target resulted in more failures, TLD ratios were different between TPS algorithms, ITV and breath hold have different treatment target extents, and the number of isocenters was not statistically significant across any of the metrics.

**Table 3.** *P* values from the Levene test for equal variances and *t* test for equal means for superior-inferior extent, TLD ratio, and GPR for both PTV1 and PTV2.

	**Pass-Fail**	**MC-PB**	**ITV-gating**	**ITV–breath hold**	**Gating–breath hold**	**1 Isocenter–2 isocenters**
PTV 1 extent						
Levene test	.99	.62	.13	.58	.34	.81
*t* test	.003^a^	.71	.44	.03^a^	.52	.40
PTV2 extet						
Levene test	.48	.67	.79	.95	.80	.64
*t* test	.002^a^	.06	.36	.06	.52	.76
PTV1 TLD ratio						
Levene test	.03^a^	.98	.37	.77	.38	.29
*t* test	<.001^a^	.03^a^	.35	.52	.80	.77
PTV2 TLD ratio						
Levene test	.44	.13	.56	.72	.74	.12
t test	.005^a^	.03^a^	.31	.28	.10	.67
PTV1 gamma pass rate						
Levene test	<.001^a^	.12	.54	.02^a^	.10	.88
*t* test	<.001^a^	.11	.90	.10	.20	.79
PTV2 gamma pass rate						
Levene test	<.001^a^	1.00	.37	.23	.14	.29
*t* test	<.001^a^	.24	.74	.11	.28	.32

**Abbreviations:** TLD, thermoluminescent dosimeter; PTV1, primary target 1; PTV2, primary target 2; MC-PB, Monte Carlo–pencil beam; ITV, internal target volume.

Note: Comparisons include pass-fail, MC-PB, motion management techniques, and number of isocenters.

a Significance was determined by α = 0.05.

The accuracy for classification random forest modeling was 73% ± 11%, while accuracy for regression was 72% ± 8%. The highest ranking variables of importance for classification and all regression variables were irradiation year and PTV1 and PTV2 extent, while the lowest ranking variables of importance were number of isocenters and TPS algorithm. **Table 4** provides the average ranking of each category for both classification and regression variables.

**Table 4.** Average ranking of variables of importance for classification and regression random forest models.

	**Classification**	**Regression**			
		**PTV1 TLD ratio**	**PTV2 TLD ratio**	**PTV1 gamma pass rate**	**PTV2 gamma pass rate**
Irradiation year	1.8 ± 0.9	2.7 ± 0.7	2.2 ± 1.1	2.7 ± 1.1	2.6 ± 1.0
PTV1 extent	1.8 ± 0.8	2.2 ± 0.4	2.7 ± 0.8	2.2 ± 0.6	1.8 ± 0.8
PTV2 extent	2.7 ± 1.2	1.1 ± 0.3	1.6 ± 0.7	1.3 ± 0.7	1.7 ± 0.7
Irradiation technique	5.1 ± 1.6	6.6 ± 1.6	4.3 ± 1.7	6.1 ± 1.9	5.4 ± 1.2
Treatment machine	5.2 ± 0.9	5.1 ± 0.7	5.0 ± 1.1	5.1 ± 1.3	4.9 ± 1.0
Motion management technique	5.6 ± 1.2	6.8 ± 1.3	6.4 ± 0.7	5.9 ± 1.1	6.6 ± 1.4
TPS	5.9 ± 1.4	4.6 ± 0.8	5.8 ± 1.0	5.2 ± 1.1	5.4 ± 1.2
Number of isocenters	8.3 ± 0.5	8.0 ± 1.5	8.7 ± 0.5	8.7 ± 0.5	8.6 ± 0.5
TPS algorithm	8.6 ± 0.7	7.9 ± 0.9	8.3 ± 0.5	7.8 ± 0.9	8.0 ± 1.2

**Abbreviations:** PTV1, primary target 1; TLD, thermoluminescent dosimeter; PTV2, primary target 2; TPS, treatment planning system.

A variety of failure modes were observed, the predominant ones being (1) systematic underdosing (no phantom suffered from overdosing), (2) poor localization in the superior-inferior direction (motion derived), and (3) range error. **Figure 3c** provides the percentage of failing phantoms per failure mode. Range error was most prominent in the anterior-posterior direction (71% and 89% for PTV1 and PTV2 failures, respectively), which aligns with the typical treatment direction and end-of-range of the beam. Only 44% of phantom failures had similar issues with both PTV1 and PTV2. For example, it was observed in several phantoms that underdosing was one of the contributions to failure modes in PTV1; however, PTV2 did not suffer from underdosing but failed owing to a different mode or combination. However, if both failed because of range errors, it was found that both targets suffered from this failure mode.

## Discussion

The pass rate for the proton liver phantom is currently the lowest of all the photon and proton phantoms provided by IROC, although aspects and trends do indicate improvement in the results with time. Correspondingly, irradiation year is a prominent variable of importance for the proton liver phantom performance. Most failures can be described by 3 different types: (1) systematic underdosing, (2) localization dose error in the direction of motion (superior-inferior), and (3) range error. Awareness and correction of these 3 errors could lead to improvement of the pass rate for the proton liver phantom and clinical treatment.

While every contributor but PTV2 TLD ratio to pass rate and the pass rate showed no statistical significance over the data cohort’s timeframe, all contributors to the pass rate and the pass rate showed an overall trend of improvement. This trend mimics expectations that experience, knowledge, and quality of proton therapy for liver treatment improve over time and lead to better treatment quality.

The error most commonly observed was systematic underdosing, which could result from incorrect beam calibration or inaccuracies in the treatment planning algorithm, such as poor modeling of multiple Coulomb scattering—which, in turn, could explain why no overdosing failure mode was observed because calculated dose is overestimated [7, 20]. Given the homogeneity of the proton liver phantom, it is surprising to observe systematic errors in dose calculation, and these errors should be further investigated by participating clinics.

Localization of dose error in the direction of motion has also been identified for the photon lung and liver phantom as a main source of error, so it is unsurprising that the proton liver phantom is prone to similar errors [11, 12]. The cause of this error could be a mismatch between alignment during simulation and irradiation of the phantom, or contouring errors in delineation of the target material from the phantom material.

Range errors can affect the distal end of the beam, causing an underdose/overdose on the edge of the target, which creates a mismatch between the film and TPS [21–23]. Issues arise with the introduction of air bubbles resultant from filling the phantom with water before simulation and again before treatment. These small air bubbles exist along the fringe of the phantom and might be missed when preparing for treatment. If they are in the path of the beam, they could cause degradation of proton beam unexpected by the TPS. While the air bubbles are characteristic of the water-filled phantom, the concept is not dissimilar to bowel gas observed in patient treatment. The presence, shape, and size of bowel gas may vary between simulation and treatment, and thus image-guided radiation therapy systems should be able to detect these differences, and proton plans should be robust enough to account for potential errors. Furthermore, the instructions provide relative stopping powers for the material making up the proton liver phantom; however, if these are miscategorized on the simulation, or setup between simulation and treatment does not align, then large enough errors to fail the phantom can occur.

According to random forest modeling, the most prominent variables of importance were the measured extent of each target in the motion direction based on the 90% isodose line; and the *t* test confirms that between pass-fail, there are differences in the means of the extent for both targets. This is also supported by the decrease in pass rate for each target as extent increases, as shown in **Figure 2b**. Breath hold has the smallest extent, while ITV has the largest extent, with static and gating having comparable extent between these two. These extents corresponded closely with pass rates and highlight that ITV motion management, despite being a relatively simple technique to implement clinically, needs further improvements to match other motion management techniques in phantom performance.

Monte Carlo had a statistically significant higher TLD ratio and gamma pass rate than pencil beam algorithms. The lack of lateral scatter modeling in the pencil beam algorithm, and the superior dosimetric accuracy of Monte Carlo, has been well documented in proton therapy of the lung [7, 24]. While the liver phantom is minimally heterogeneous, the superiority of Monte Carlo is nevertheless apparent.

Upon completion of the phantom irradiation, clinics are provided with a report that includes the TLD ratios, gamma pass rate for all planes, and representative line profiles through the target in all 3 directions. With this report and, when possible and necessary, guidance from the physics staff at IROC, strategies are identified on possible ways to improve performance. Many institutions (∼50%) do successfully rectify issues and pass on their subsequent attempt. However, many institutions required a large number of attempts to successfully irradiate the phantom. The inability to resolve problems more efficiently highlights the limitations of an end-to-end test: it is very difficult to troubleshoot when things go wrong. The proton liver phantom involves several challenging components, and repeated irradiations often resulted in different failure modes.

Owing to the limited number of clinics that perform proton therapy and the complex nature of the proton liver phantom, our data set was relatively small and some constituents did not have the power needed to determine significance or provide proper influence for our random forest modeling. However, this cohort is representative of the current practice and can shed light on inadequacies in the clinical workflow pertaining to the proton liver phantom and the important factors that might help improve overall pass rates.

## Conclusions

While improvement during the last 4 years has been observed, more work is needed to increase the pass rate of proton liver phantom, which has a historical average of 52%. As seen in clinical trials assessing proton therapy for liver malignancies, range uncertainty and motion management play a large role in determining phantom performance. In addition, systematic underdosing was also a prominent failure mode for the proton liver phantom, which could be the result of inaccuracies in the dose algorithm.

To improve performance in delivering this type of treatment, careful use of pencil beam algorithms and ITV motion management techniques is needed, as these lag behind other algorithms and techniques in terms of dose accuracy. Careful consideration of the influences affecting liver proton therapy can help maximize not only phantom performance but also patient outcome.
